# Enhancement of Vaccine-Induced T-Cell Responses by Probiotics in Calves

**DOI:** 10.3390/vaccines13111120

**Published:** 2025-10-31

**Authors:** Mari Ikehata, Tomohiro Okagawa, Hayato Nakamura, Naoya Maekawa, Yasuhiko Suzuki, Shiro Murata, Kazuhiko Ohashi, Satoru Konnai

**Affiliations:** 1 Department of Disease Control, Faculty of Veterinary Medicine, Hokkaido University, Sapporo 060-0818, Japan; 2 Business Development Unit, FASMAC Co., Ltd., Atsugi 243-0021, Japan; 3 Institute for Vaccine Research and Development, Hokkaido University, Sapporo 001-0021, Japan; 4 Veterinary Research Unit, International Institute of Zoonosis Control, Hokkaido University, Sapporo 001-0020, Japan; 5 International Affairs Office, Faculty of Veterinary Medicine, Hokkaido University, Sapporo 060-0818, Japan; 6 One Health Research Center, Hokkaido University, Sapporo 060-0818, Japan

**Keywords:** probiotics, *Clostridium butyricum*, vaccine, growth rate, T cell, calf

## Abstract

Background/Objectives: Calves have immature immune systems, hence immunization with vaccines is essential to protect them from infectious diseases. However, immune responses to vaccines vary widely among individuals. Therefore, strategies for enhancing vaccine efficacy are needed, particularly those targeting low responders to vaccines. Probiotics have attracted attention because of their beneficial immunomodulatory effects on the host. Although probiotics may improve calf immunity, their potential to enhance immune responses to vaccines in calves remains unclear. Thus, we investigated whether immune responses to vaccines, especially T-cell responses, are enhanced when calves receive a combination of probiotic supplementation and vaccination. Methods: Calves were divided into three feeding groups, as follows: negative control feed, live bacteria-mixed feed (Zeosapo KB), and *Clostridium butyricum*-only feed (CB). After weaning, all calves received two doses of a live attenuated hexavalent viral vaccine. T-cell responses to a vaccine antigen were evaluated by measuring the expression levels of lymphocyte activation markers CD25 and CD69, as well as Th1 cytokine production, in peripheral blood mononuclear cell culture assays. Results: CD25 expression significantly increased in CD4^+^ T cells four weeks after the booster vaccination in the Zeosapo KB- and CB-fed groups. In addition, the CD25^+^CD69^+^ cell ratio in CD4^+^ T cells was increased in these groups. The production of Th1 cytokines in the culture supernatant was also increased in the CB-fed group. Conclusions: This clinical study demonstrates that probiotics activate CD4^+^ T cells and enhance Th1 cytokine responses in vaccinated calves.

## 1. Introduction

Vaccination is one of the most effective methods of controlling infectious diseases by safeguarding individuals from disease and death and limiting the propagation of infections within communities. Various vaccines have been developed in the field of veterinary medicine to control infectious diseases in animals. Vaccination plays a vital role in preventing infections among livestock maintained in group housing systems. Neonatal animals, such as calves, have immature immune systems, making them susceptible to various infectious diseases, including diarrhea and respiratory infections. Therefore, immunization by vaccination is essential to protect calves from infectious diseases. Live attenuated vaccines are the most widely used in vaccinating livestock, inducing both cellular and humoral immunity [1]. However, responses to vaccines vary significantly among individuals, thereby inducing varying levels of immunity. Therefore, strategies must be developed to enhance the protective efficacy of vaccines, particularly among vaccine low responders.

‘Probiotics’ refers to living microorganisms that support host health when consumed in sufficient quantities. Various strains of bacteria, including lactic acid bacteria and butyric acid bacteria, are currently used as probiotics in food supplements for humans and animals, including calves. A variety of clinical benefits linked to probiotic administration have been reported in human studies. In rural India, where infectious diseases still result in significant infant mortality, a previous study reported the use of lactic acid bacteria as a preventive intervention in newborns [2]. Furthermore, in veterinary medicine, a previous study demonstrated that feeding fermented milk replacer (MR) and MR supplemented with highly concentrated *Lactiplantibacillus plantarum* HOKKAIDO strain (Lp-HKD) mitigated bovine rotavirus-induced diarrhea and attenuated intestinal tissue in neonatal calves [3]. Because of their various beneficial effects on the host, probiotics are used widely, not only for humans but also for animals.

The role of probiotics in modulating host immune responses has garnered significant attention because of their impact on immune cell signaling pathways and differentiation, and their role in enhancing both innate and acquired immune responses [4]. Therefore, probiotics may influence immune responses to vaccines. Several studies on gnotobiotic piglets with human intestinal microbiota vaccinated with oral attenuated human rotavirus (HRV) demonstrate that *Lactobacillus rhamnosus* GG strain administration enhances HRV-specific immune responses, including interferon (IFN)-γ producing T cell, and elevates levels of immunoglobulin A (IgA) and IgG antibodies [5,6]. Furthermore, in broiler chickens, the combination of *Clostridium butyricum* administration with avian coccidiosis vaccination significantly enhanced growth performance, and also reduced fecal oocysts shedding following *Eimeria* sp. infection [7]. *C*. *butyricum* are one of the strains of non-toxigenic butyric acid bacteria, and have been studied as probiotic bacteria in humans [8]. Holstein heifers fed with *C*. *butyricum* LXKJ-1 strain showed improved growth rates [9]. A previous study also reported that the oral administration of *C*. *butyricum* MIYAIRI 588 strain prevented the decrease in CD4^+^ T-cell numbers during the periparturient period in cows [10]. In addition to *C*. *butyricum*, we found that Lp-HKD also exhibited both immunostimulatory and antiviral effects while modulating bovine immune responses to viral infections [11]. Thus, probiotics can improve immunity in calves. However, the effect of probiotics on the immune responses to vaccines in calves has not been demonstrated.

In this study, we investigated whether probiotics enhance calf immune response, especially T-cell responses, to vaccines in a large calf population. Calves were vaccinated twice with a hexavalent vaccine against viruses causing bovine respiratory infections during probiotic feeding (live bacteria-mixed feed or *C*. *butyricum* alone). To evaluate T-cell responses to vaccination in calves, changes in the numbers of viral antigen-responding T cells and cytokine response were examined. Furthermore, changes in growth rate were also assessed.

## 2. Materials and Methods

### 2.1. Experimental Design and Sample Collection

**Figure 1:**
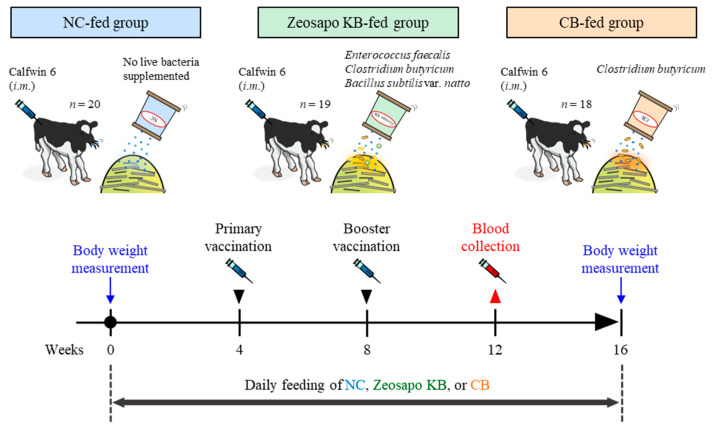
Outline of a clinical study of vaccination during feeding of NC, Zeosapo KB, or CB. This clinical study was divided into three feeding groups: (1) twenty calves fed with negative control feed (NC); (2) nineteen calves fed with live bacteria-mixed feed (Zeosapo KB); and (3) eighteen calves fed with *C*. *butyricum*-only feed (CB). All calves were administered with “KYOTOBIKEN” Calfwin 6 Combo Live Vaccine intramuscularly at 4 and 8 weeks after the start of the feeding. Peripheral blood samples were collected from all calves 4 weeks after the booster vaccination. The calves were weighed at the start and end of the study. i.m.–intramuscular injection. The date for each treatment is shown in Supplemental Table S2.

The design of the animal experiment is shown in Figure 1. Two-month-old male Holstein calves (Supplemental Table S1) were divided into three feeding groups: negative control feed (NC), live bacteria-mixed feed (Zeosapo KB; a commercial feed product form Kyoto Biken Laboratories, Uji, Japan), and *C. butyricum* (CB)-only feed (Kyoto Biken Laboratories). The first group (*n* = 20) was fed NC, which was supplemented only by rice bran wax (71.5%) and zeolite (28.5%). The second group (*n* = 19) was fed Zeosapo KB, which is a mixed feed supplemented with the following three types of live bacteria: *Enterococcus faecalis* NT strain (strain ID: NBRC 100481), *C*. *butyricum* NT strain (strain ID: NBRC 13949), and *Bacillus subtilis* var. *natto* NT strain (strain ID: GTC 02853). In addition, the Zeosapo KB was supplemented with rice bran wax (61.5%), zeolite (28.5%), and isomalto-oligosaccharide (10.0%). The colony forming units (CFUs) for each strain were quantified using the serial dilution and plate count method. The calf feed was supplemented with each strain at 3 × 10^7^ CFU/calf/day according to the feeding protocol provided by the manufacturer. The third group (*n* = 18) was fed *C. butyricum* NT strain (3 × 10^9^ CFU/calf/day), a concentration one hundred times higher than that of the Zeosapo KB-fed group, along with rice bran wax (71.5%) and zeolite (28.5%). All feeds used in this study were kept in a storage shed protected from sunlight and moisture. Each feed was stored in paper bags and used entirely within four weeks of opening. All three groups were administered their respective feeds every day. All calves received two intramuscular administrations of the hexavalent live attenuated vaccine “KYOTOBIKEN” Calfwin 6 Combo Live Vaccine (Kyoto Biken Laboratories) at four and eight weeks after the start of the feeding. This vaccine contains the following viral components: bovine respiratory syncytial virus (BRSV, rs-52 strain, >10^5.0^ TCID_50_), bovine viral diarrhea virus (BVDV) type 1a (No. 1255 strain, >10^3.0^ TCID_50_), BVDV type 2 (KZ1254 strain, >10^3.0^ TCID_50_), bovine herpesvirus-1 (BHV-1, No. 758-43 strain, >10^4.0^ TCID_50_), parainfluenza virus type 3 (PIV3, BN-CE strain, >10^5.0^ TCID_50_), and adenovirus type 7 (TS-GT strain, >10^3.0^ TCID_50_). At four weeks post-booster vaccination, peripheral blood samples were collected from all calves. Peripheral blood mononuclear cells (PBMCs) were isolated from blood samples via density gradient centrifugation using Percoll (GE Healthcare, Little Chalfont, UK). Body weights were measured twice, at the start and end of the feeding. Weight gain was calculated as follows:

weight gain (%) = (weight at the end of the feeding − weight at the start of the feeding)/weight at the start of the feeding × 100

All experimental procedures were carried out with the approval of the Ethics Committee of the Faculty of Veterinary Medicine, Hokkaido University (approval number: 22-0038; approval date: 23 March 2022). All procedures in this study adhered to the ARRIVE guidelines [12].

### 2.2. PBMC Cultivation Assay

T-cell responses to vaccination were analyzed using a cultivation assay of PBMCs. Fresh PBMCs (1 × 10^6^) were cultured with 1.95 × 10^4^ 50% tissue culture infectious dose (TCID_50_)/mL of UV-inactivated BRSV (rs-52 strain) or 0.5 µg/mL of concanavalin A (ConA; Sigma-Aldrich, St. Louis, MO, USA) in duplicate in 96-well round-bottom microplates (Corning Inc., Corning, NY, USA) containing 200 µL Roswell Park Memorial Institute 1640 Medium (Sigma-Aldrich) containing 10% heat-inactivated fetal bovine serum (Thermo Fisher Scientific, Waltham, MA, USA), 200 IU/mL penicillin (Thermo Fisher Scientific), 200 µg/mL streptomycin (Thermo Fisher Scientific), and 0.01% L-glutamine (Thermo Fisher Scientific) at 37 °C under 5% CO_2_ for 5 days. PBMCs cultured without stimulation were prepared as the negative control. Following the cultivation, cells were collected for flow cytometric analysis, and culture supernatants were collected and stored at −30 °C.

### 2.3. Flow Cytometric Analysis of T Cells

To evaluate T-cell responses to vaccination, cultured PBMCs were analyzed for CD25 and CD69 expression by flow cytometry. The cultured PBMCs were harvested and blocked with phosphate-buffered serum (PBS) containing 10% goat serum (Thermo Fisher Scientific) at 25 °C for 15 min. After washing, the cells were stained with PerCp/Cy5.5-conjugated anti-CD3 monoclonal antibody (mAb; MM1A; Washington State University Monoclonal Antibody Center, Pullman, WA, USA), PE/Cy7-conjugated anti-CD4 mAb (CC30; Bio-Rad, Hercules, CA, USA), PE-conjugated anti-CD8 mAb (CC63; Bio-Rad), Alexa Fluor 488-labeled anti-CD25 mAb (CACT116A; VMRD, Pullman, WA, USA), Alexa Fluor 647-labeled anti-CD69 mAb (KTSN7A; Washington State University Monoclonal Antibody Center), and Fixable Viability Dye eFluor 780 (Thermo Fisher Scientific) at 4 °C for 20 min. MM1A and CC30 were conjugated with PerCp/Cy5.5 and PE/Cy7, respectively, using Lightning-Link Conjugation Kits (Abcam, Cambridge, UK). CACT116A and KTSN7A were prelabeled using the Zenon Alexa Fluor 488 and Zenon Alexa Fluor 647 Mouse IgG_1_ Labeling Kits (Thermo Fisher Scientific), respectively. Antibody dilution and cell washing were performed with PBS containing 1% bovine serum albumin (Sigma-Aldrich) and 2 mM ethylenediaminetetraacetic acid (Dojindo Molecular Technologies, Kumamoto, Japan). The stained cells were washed and analyzed immediately using the FACSLyric Flow Cytometry System (BD Biosciences, San Jose, CA, USA).

### 2.4. Quantification of Cytokines by Enzyme-Linked Immunosorbent Assay

To examine T-cell responses to vaccination in culture supernatants of PBMCs, IFN-γ and tumor necrosis factor (TNF)-α concentrations were analyzed by enzyme-linked immunosorbent assay (ELISA). Culture supernatants of PBMCs were collected, and IFN-γ and TNF-α concentrations were measured using the Bovine IFN-γ ELISA Development Kit (Mabtech, Nacka Strand, Sweden) and Bovine TNF-α ELISA Development Kit (Mabtech), respectively, according to the manufacturers’ protocols.

### 2.5. Statistical Analysis

Differences were determined using the Dunn’s test after the Kruskal–Wallis test for multiple-group comparisons using GraphPad Prism 10.1.0 (GraphPad Software, San Diego, CA, USA). A *p*-value < 0.05 was considered statistically significant. No correction was applied for multiple endpoints as this study is exploratory.

## 3. Results

### 3.1. Enhancement of Weight Gain by Feeding of Zeosapo KB and CB

**Figure 2:**
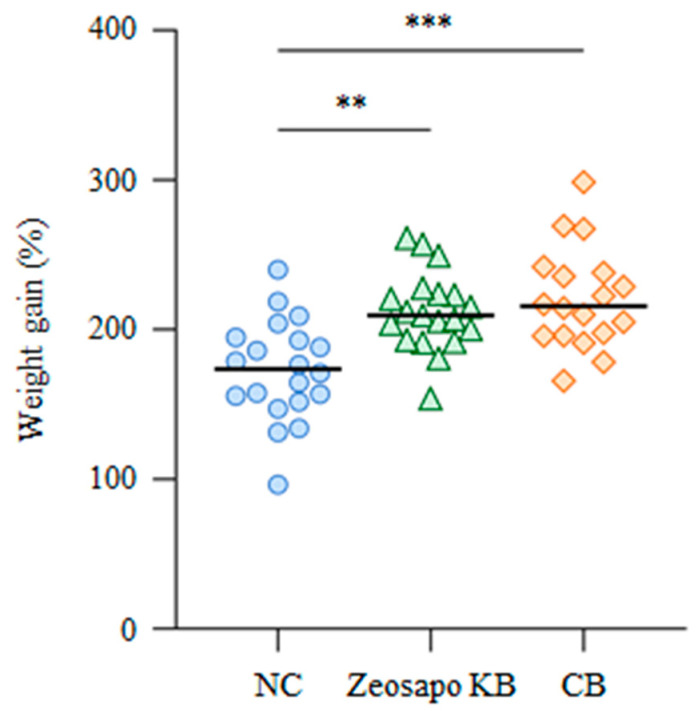
Effects of Zeosapo KB or CB feeding on growth rate. The body weights of all calves were measured at the start and end of the feeding (NC, circle, *n* = 20; Zeosapo KB, triangle, *n* = 19; CB, diamond, *n* = 18). Weight gain (%) was calculated as (weight at the end of the feeding − weight at the start of the feeding)/weight at the start of the feeding × 100. Statistical significance was determined using the Dunn’s test after the Kruskal–Wallis test for multiple group comparisons. ** *p* < 0.01, *** *p* < 0.001.

To examine the effects of Zeosapo KB and CB on the growth rate of calves, all calves were weighed individually at the start and end of the feeding. The median weight gains of the NC-, Zeosapo KB-, and CB-fed groups were 173.7, 209.6, and 215.7%, respectively (Figure 2). These results suggest that a more significant weight gain was observed in the Zeosapo KB- and CB-fed groups than the NC-fed group.

### 3.2. Activation of Whole T-Cell Subsets by CB Feeding

**Figure 3:**
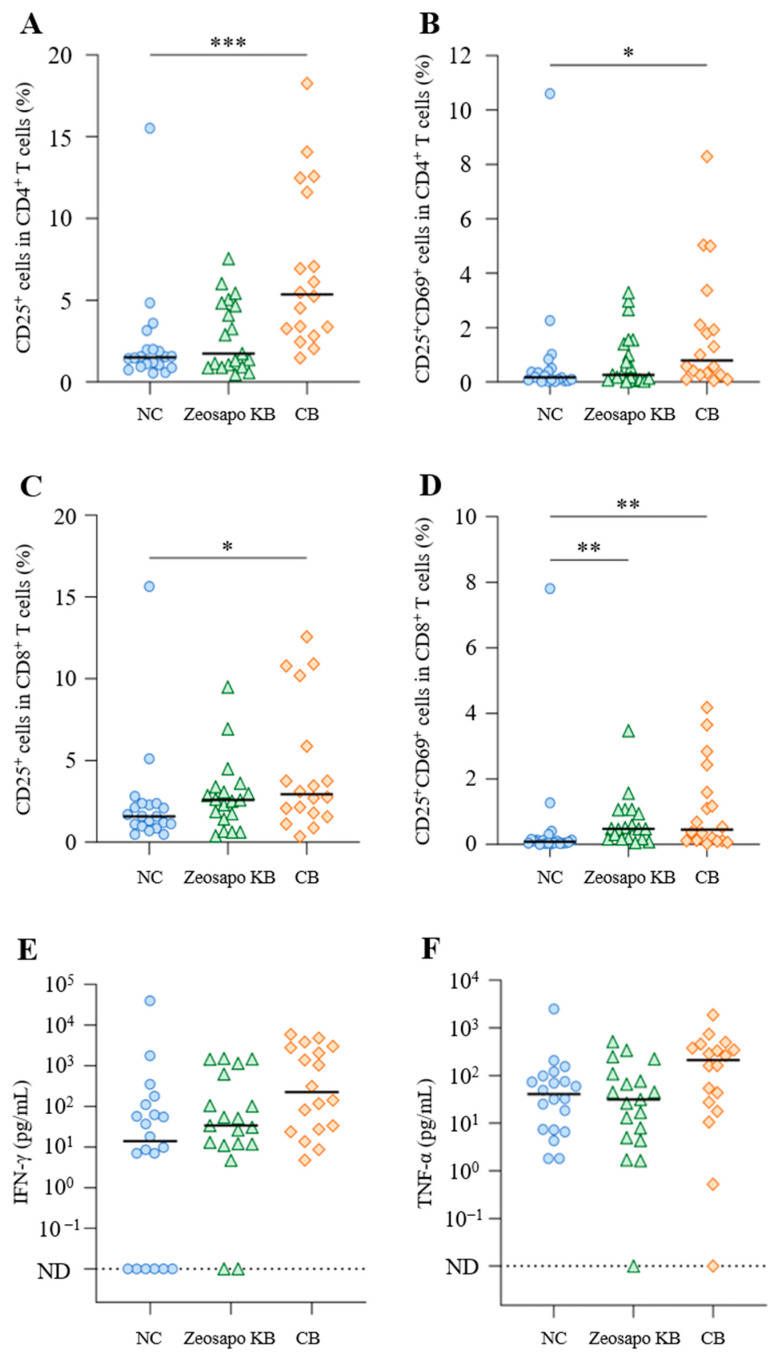
Effects of Zeosapo KB or CB feeding on T-cell subsets in PBMCs. (**A**–**F**) PBMCs isolated from calves (NC, circle, *n* = 20; Zeosapo KB, triangle, *n* = 19; CB, diamond, *n* = 18) 4 weeks after the booster vaccination were cultured only with medium for 5 days. The proportions (%) of CD25^+^ and CD25^+^CD69^+^ cells in CD3^+^CD4^+^ T cells (**A**,**B**) and CD3^+^CD8^+^ T cells (**C**,**D**) were analyzed by flow cytometry. IFN-γ (**C**) and TNF-α (**D**) productions from PBMCs isolated from each animal were measured by ELISA in duplicate. Statistical significance was determined by the Dunn’s test after the Kruskal–Wallis test for multiple-group comparisons. * *p* < 0.05, ** *p* < 0.01, *** *p* < 0.001. ND, not detected. The data for IFN-γ and TNF-α included samples that were below the detection limit and the statistical analysis was not performed on these data.

To determine the effects of Zeosapo KB and CB feeding on whole T-cell subsets, we compared T-cell activation without stimulation among the three groups. Four weeks after the booster vaccination, CD25 and CD69 expression was analyzed as activation-induced markers (AIM) [13] in CD3^+^CD4^+^ and CD3^+^CD8^+^ T cells from PBMCs cultured without stimulation by flow cytometry (Figures S1–S3). The percentages of CD25^+^ and CD25^+^CD69^+^ cells in both CD3^+^CD4^+^ and CD3^+^CD8^+^ T cells were significantly increased in PBMCs under CB feeding (Figure 3A–D). Furthermore, the percentage of CD25^+^CD69^+^ cells in CD3^+^CD8^+^ T cells significantly increased in the Zeosapo KB-fed group (Figure 3D). Furthermore, we also measured IFN-γ and TNF-α levels in supernatants of PBMCs cultured without stimulation four weeks after the booster vaccination by ELISA. IFN-γ production was significantly higher in the CB-fed group (median: 225.1 pg/mL) than in the NC-fed group (median: 13.8 pg/mL; Figure 3E). TNF-α production also tended to increase in the CB-fed group (Figure 3F). The median TNF-α concentration was 40.8 and 211.5 pg/mL in PBMCs cultured without stimulation in the NC- and CB-fed groups, respectively (Figure 3F). These results indicate that the oral feeding of CB could activate T cells and induced Th1 cytokine production in circulating PBMCs.

### 3.3. Activation of Vaccine-Specific CD4^+^ T-Cell Responses in Zeosapo KB- and CB-Fed Groups

**Figure 4:**
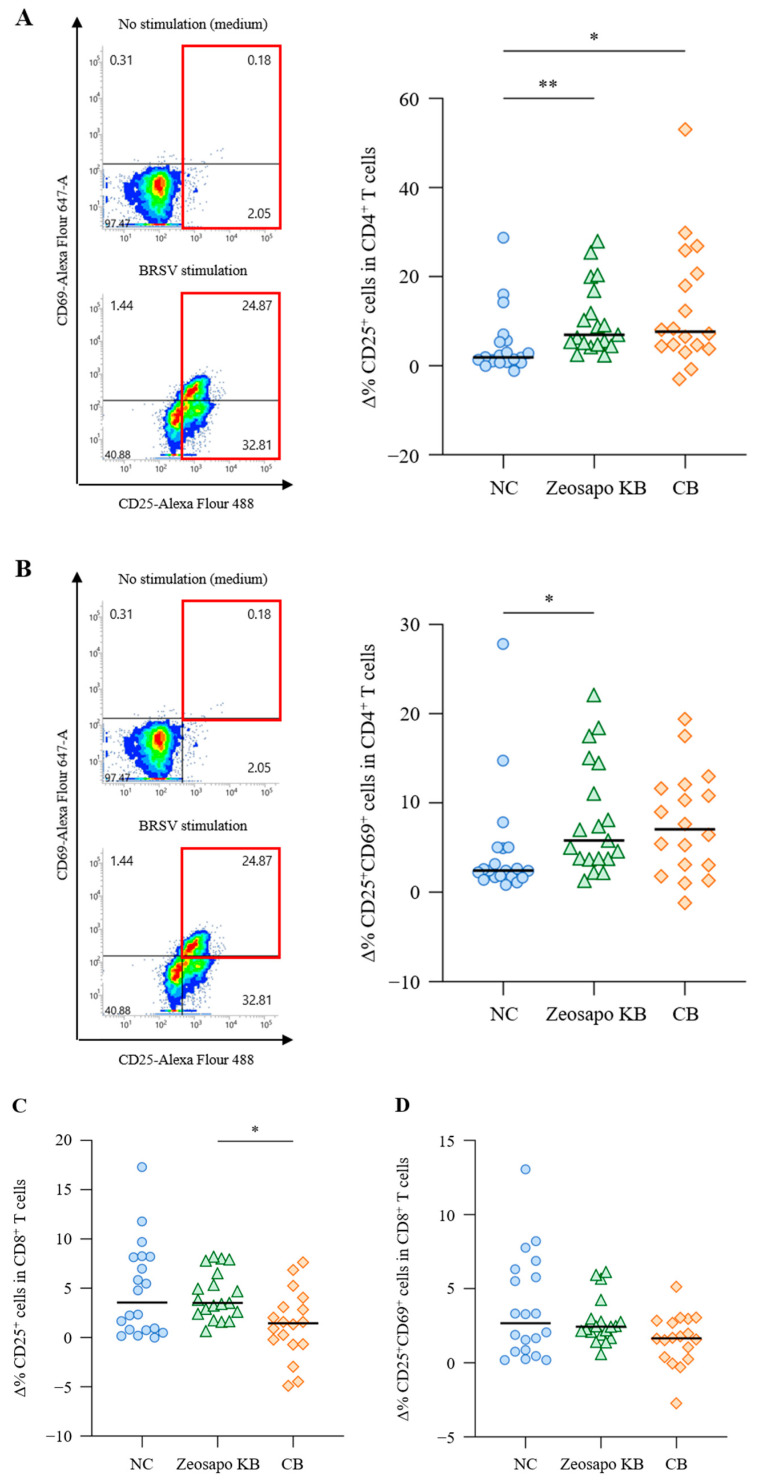
Activation of CD4^+^ T-cell responses to BRSV antigen. (**A**–**D**) PBMCs isolated from calves (NC, circle, *n* = 20; Zeosapo KB, triangle, *n* = 19; CB, diamond, *n* = 18) 4 weeks after the booster vaccination were cultured with or without BRSV antigen for 5 days. The proportion (%) of CD25^+^ and CD25^+^CD69^+^ cells in CD3^+^CD4^+^ and CD3^+^CD8^+^ T cells was analyzed by flow cytometry. The percent changes in CD25^+^ T cells (**A**,**C**) and CD25^+^CD69^+^ T cells (**B**,**D**) were assessed using the equations shown above. Statistical significance was determined using the Dunn’s test after the Kruskal–Wallis test for multiple-group comparisons. * *p* < 0.05, ** *p* < 0.01.

To evaluate the effects of Zeosapo KB and CB on vaccine-specific immune responses, T-cell responses to BRSV antigen were analyzed four weeks after the booster vaccination. PBMCs were stimulated with BRSV antigen, and CD25 and CD69 expressions as AIM on T cells were analyzed by flow cytometry (Figure 4A). CD3^+^CD4^+^ and CD3^+^CD8^+^ T cells were gated in live lymphocytes and then analyzed for CD25 and CD69 expression (Figures S1–S3). The Δ% CD25^+^ T cells and Δ% CD25^+^CD69^+^ T cells were determined to evaluate vaccine-specific T-cell responses as follows:

Δ% CD25^+^ T cells = % CD25^+^ T cells (BRSV antigen) − % CD25^+^ T cells (medium)

Δ% CD25^+^CD69^+^ T cells = % CD25^+^CD69^+^ T cells (BRSV antigen) − % CD25^+^CD69^+^ T cells (medium)

The Δ% CD25^+^ cells were significantly higher in CD3^+^CD4^+^ T cells stimulated with BRSV antigen in the Zeosapo KB- and CB-fed groups than in those in the NC-fed group (Figure 4A). Furthermore, the Δ% CD25^+^CD69^+^ cells significantly increased in CD3^+^CD4^+^ T cells stimulated with BRSV antigen, especially in the Zeosapo KB group (Figure 4B). However, CD8^+^ T-cell activation was not strongly induced, even after the booster vaccination (Figure 4C,D). Thus, the proportion of activated CD4^+^ T cells in response to BRSV antigen increased in the Zeosapo KB- and CB-fed groups.

### 3.4. Enhancement of Vaccine-Induced Th1 Cytokine Responses by CB Feeding

**Figure 5:**
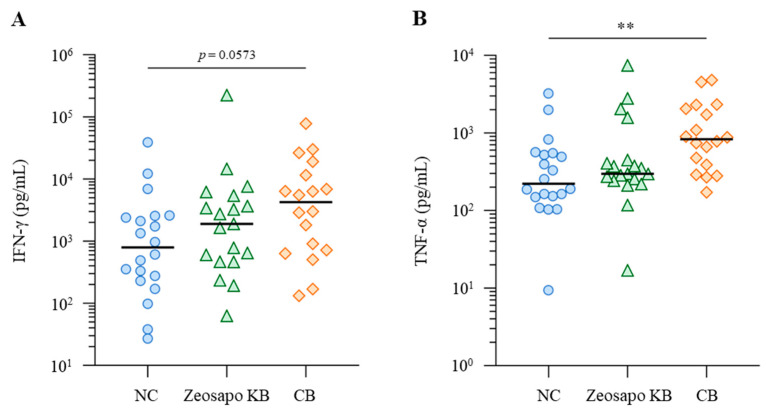
Activation of Th1 cytokine responses to BRSV antigen. (**A**,**B**) PBMCs isolated from calves (NC, circle, *n* = 20; Zeosapo KB, triangle, *n* = 19; CB, diamond, *n* = 18) 4 weeks after the booster vaccination were cultured in the presence of BRSV antigen for 5 days. IFN-γ (**A**) and TNF-α (**B**) production from PBMCs isolated from each animal was measured by ELISA in duplicate. Statistical significance was determined using the Dunn’s test after the Kruskal–Wallis test for multiple-group comparisons. ** *p* < 0.01.

The Th1 cytokine’s response to vaccination is evaluated as a functional parameter of T cells. In this study, we assessed Th1 immunity by measuring the antigen-induced production of IFN-γ and TNF-α. PBMCs collected from calves four weeks after the booster vaccination were cultured in the presence of BRSV antigen, with IFN-γ and TNF-α productions measured by ELISA. Among the three groups, IFN-γ responses to BRSV antigen tended to increase, especially in the CB-fed group (Figure 5A). The median IFN-γ concentrations in BRSV-stimulated PBMCs were 789.6, 1892.6, and 4252.13 pg/mL for the NC-, Zeosapo KB-, and CB-fed groups, respectively (Figure 5A). Furthermore, the TNF-α production in culture supernatants was significantly higher in the CB-fed group (median: 828.2 pg/mL) than in the NC-fed group (median: 222.0 pg/mL; Figure 5B). Overall, Th1 cytokine responses to BRSV antigen were more strongly induced in the CB-fed group than in the NC-fed group.

## 4. Discussion

Calf pneumonia is a major concern in the cattle industry, contributing to substantial economic losses globally due to growth retardation in surviving calves and occasional mortality [14,15]. In general, the pathogenesis of respiratory disease in calves involves a complex interplay of host defense, the stress-induced impairment of respiratory protection, and concurrent primary infection with respiratory viruses, such as BRSV, BVDV, and PIV3 [16,17]. Viral infection and the ensuing host immune response further compromise host defense, possibly triggering bacterial infections in pulmonary tissues, leading to death or lifelong growth failure in infected calves [16,17]. BRSV is one of the common infectious viral agents that is closely related genetically and antigenically to human RSV (HRSV), a major cause of respiratory infections in infants [18,19]. Therefore, BRSV infection in calves is an excellent animal model for investigating the pathogenesis and immunological mechanisms of HRSV infection [18,19]. To control respiratory infections in calves, multivalent vaccines targeting various viral pathogens have been developed and incorporated into vaccination programs for calves [20,21,22,23]. However, responses to vaccines vary among individuals, and not all calves can develop sufficient protective immune responses to infection. Hence, even after repeated vaccinations, some calves remain at risk of infection. Therefore, strategies must be developed to elicit more robust immune responses to vaccines, particularly in such individuals.

Because probiotics are considered immune response modifiers, their use may strengthen the efficacy of vaccination [23]. Human studies have demonstrated the ability of probiotics to enhance the protective efficacy of vaccines [24]. The effects of probiotics on enhancing immune responses to vaccines have also been investigated, mainly in pigs and chickens [23]. Pigs and piglets were used in animal models for evaluating the potential augmentative effect of probiotics on vaccines against human viruses [25,26,27]. Since chickens are also a source of human epidemic infections, the use of probiotics to improve vaccine efficacy has been widely investigated in avian models [23]. However, few reports have investigated in immunological detail whether the feeding of probiotics augments vaccine efficacy in calves. Therefore, this study examined the clinical efficacy of probiotics for boosting vaccine-specific immunity in a large study population of calves.

We evaluated vaccine-specific T-cell responses using AIM assays and cytokine production measurements. AIM assays are widely used as an accessible and rapid method for determining vaccine efficacy by measuring multiple markers of T-cell responses [13]. Other methods can be used to detect antigen-specific T cells by measuring cytokine production, such as intracellular cytokine staining [28]. However, cytokines are produced with different kinetics; hence, it is hard to detect intracellular cytokines by flow cytometry at any given timepoint [13]. Numbers of AIM^+^ T cells have been shown to correlate with numbers of cytokine-producing T-cell responses to vaccine antigens, so AIM assays can provide a broader view of T-cell responses to vaccines [29]. As for cytokine production, we measured IFN-γ and TNF-α as representative cytokine-produced effector T cells by using ELISA instead of intracellular cytokine staining. Although AIM assays are not widely used in bovine immunology, this technique is expected to have utility in future studies, and we also followed the previous study, which used AIM assays to study T-cell responses to vaccination in cattle [30]. BRSV is known to be the most potent inducer of cellular immune responses among the six antigens included in the hexavalent vaccine [31]. Thus, we used the BRSV antigen for the PBMC stimulation assays. On the other hand, the other viruses (BVDV, BHV-1, PIV3, and adenovirus) predominantly induce humoral responses, and their antigen-specific cellular responses are estimated to be weak or undetectable in PBMC assays [32,33,34,35]. In this study, we used the BRSV antigen among viral antigens in the hexavalent vaccine in cultivation assays because the BRSV antigen was especially suitable for the stimulation of immune cells in these in vitro assays.

To ensure comprehensive immunity against BRSV, the induction of T-cell responses in addition to humoral responses is considered essential [20]. In this study, we measured the level of the neutralizing antibody (NAb) to BRSV at the beginning of the study. However, some of the calves showed high titers of maternally-derived anti-BRSV NAb before vaccination (Supplemental Table S1). Hence, changes in vaccine-induced NAb titers were not assessed in this study. Instead of humoral responses, we evaluated cellular-mediated immunity by use of AIM assays and cytokine measurements. In the context of RSV infection, IFN-γ and TNF-α are key molecules that mediate the antiviral Th1 responses, activating natural killer cells and cytotoxic CD8^+^ T cells to promote viral clearance and recovery from a viral challenge in calf and mouse models [36,37,38,39,40,41]. However, in terms of the results of CD8^+^ T-cell responses to the BRSV antigen in the present study, there was no significant difference among the three groups compared. As for the previous study [29], CD8^+^ T-cell responses to the BRSV antigen tended to be lower than CD4^+^ T-cell responses to that antigen in this in vitro assay. It may be possible to accurately evaluate the CD8^+^ T-cell response by using optimal stimulation methods for CD8^+^ T cells, such as co-culture assays with antigen-pulsed antigen-presenting cells [42].

We observed that CD4^+^ T-cell activation and cytokine responses were significantly enhanced four weeks after the booster vaccination in Zeosapo KB-fed and CB-fed groups. In human and mice studies, the mechanisms by which probiotics ameliorate vaccine efficacy have been well investigated [43]. Several reports have indicated that gut microbiota impact vaccine-specific immune responses and promote the induction of nonspecific responses via the training of innate immune cells [43]. Cai et al. [7] revealed that changes in the gut microbiota diversity of broiler chickens by feeding with *C*. *butyricum* enhanced the protective effects of vaccination. However, in the present study, we cannot elucidate the mechanisms behind the effects of probiotics on enhancing vaccine efficacy in calves. To determine the mechanisms underlying the enhancement of T-cell responses when following the vaccination with the feeding of probiotics, more detailed analyses are warranted. Furthermore, in this study, although the induction of T-cell responses to the vaccine antigen tended to be higher in the CB-fed group compared with the Zeosapo KB-fed group, there was no significant difference between these groups. The total number of bacteria in Zeosapo KB is not same as that in CB feed, but Zeosapo KB contains isomalto-oligosaccharide as prebiotics. A previous report comparing the efficacy of single-strain probiotics and multi-strain probiotic mixtures in humans confirmed that single strains demonstrated equivalent effects to mixtures in most cases [44]. However, it remains uncertain whether the feeding of a single strain or multiple strains to calves provides equivalent or superior effects in enhancing vaccine efficacy. The CB-fed group received 100-fold higher doses of *C. butyricum* than the Zeosapo KB-fed group. Therefore, it cannot be ruled out that the observed effects may be dose-dependent for *C. butyricum*. This represents a limitation of the present study, and further investigation is needed to clarify the dose-dependent effects of probiotics in calves. In addition, numbers of treatments for the respiratory disease in the Zeosapo KB-fed and CB-fed groups tended to be fewer than in the NC-fed group (Supplemental Table S3), although the number of animals that developed the disease was too small to allow for the necessary statistical analysis. The present study did not evaluate the protective efficacy of the combinatorial use of vaccination and probiotic feeding under viral challenge conditions. Therefore, further experiments of viral challenge are warranted to clarify the role of probiotics in enhancing the effects of vaccines in calves.

Furthermore, four months of oral feeding with Zeosapo KB or CB improved the growth rates of calves. The body weight gains in calves during this study in the Zeosapo KB- and CB-fed groups were significantly higher than those in the NC-fed group. Several reports have shown that *C*. *butyricum* supplements improved rumen fermentation and intestinal health, and elevated growth performance in ruminant animals, such as cattle and goats [9,45,46,47]. In this study, we did not evaluate the effects of *C*. *butyricum* on rumen fermentation and gut microbiota in calves, but the modulation of rumen and gut microbiota by *C*. *butyricum* administration may have led to the improvement of growth performance in calves. The CB-fed group showed better weight gain than the Zeosapo KB-fed group, suggesting that the concentration of *C*. *butyricum* administration might play a role in growth performance in calves. Furthermore, the oral feeding of Zeosapo KB or CB could induce a nonspecific but enhanced immune state in calves. Magalhães et al. [48] examined the interaction between the feeding of probiotics and the function of PBMCs in healthy calves, and revealed that orally administered probiotics, consisting of *Lactobacillus plantarum*, *Enterococcus faecium*, and *C*. *butyricum*, increased the numbers of CD4^+^ and CD8^+^ T cells and elevated the expressions of IFN-γ and TNF-α in peripheral leukocytes in calves. Focusing on *C*. *butyricum*, a previous study revealed that the oral administration of the *C*. *butyricum* MIYAIRI 588 strain in mice infected with *Clostridioides difficile* promoted the differentiation of IFN-γ-producing Th1 cells [49]. In this study, nonspecific T-cell activation in the CB-fed group, which was fed a high concentration of *C*. *butyricum*, was higher than that in the Zeosapo KB-fed group, and so it was suggested that *C*. *butyricum* mainly enhanced nonspecific T-cell responses in calves. Taken together, *C*. *butyricum* supplemented into feed could improve the growth rate and promote cellular immune function in healthy calves.

## 5. Conclusions

The results of this clinical study demonstrate that probiotics increase activated CD4^+^ T cells and induce Th1 cytokine production in response to antigens in vaccinated calves. Therefore, probiotics are a promising candidate for enhancing the protective efficacy of vaccines, thereby contributing to the reduction in infectious diseases and antibiotic use in calves. Furthermore, the results of the present study indicate that the use of *C*. *butyricum* enhanced the growth rate and nonspecific immune response of calves. Further studies are necessary to understand the mechanisms involved in the enhanced immune response to vaccines induced by the feeding of probiotics. Viral challenge experiments should be conducted to further determine whether probiotics enhance vaccine efficacy.

## 6. Patents

A patent application is pending for the materials and techniques presented in this paper (applicant, S.K., T.O., N.M., S.M., K.O., M.I., and H.N.; application number, 2024-231991).

## Data Availability

The original contributions presented in this study are included in the article/Supplementary Materials. Further inquiries can be directed to the corresponding author.

## Supplementary Materials

The following supporting information can be downloaded at: https://www.mdpi.com/article/10.3390/vaccines13111120/s1, Figure S1: Gating strategy and representative dot plots for CD25 and CD69 expression in vaccinated calves; Figure S2: Response of CD8^+^ T cells against BRSV antigen stimulation; Figure S3: Effects of Zeosapo KB or CB feeding on T-cell subsets in PBMCs; Table S1: Information of individual animals at the start of feeding; Table S2: Dates of the treatments in this study; Table S3: Numbers of the treatments for the respiratory disease during this study.
