# Supplementary material for: Enhancement of Vaccine-Induced T-Cell Responses by Probiotics in Calves
Source: Vaccines (Basel). 2025 Oct 31;13(11):1120. doi: 10.3390/vaccines13111120 (PMC12656743; doi:10.3390/vaccines13111120)
Supplement: Supplementary file 1 [file vaccines-13-01120-s001.zip › Supplemental materials/Supplemental Figures (Iketaha et al)_revision.pptx]

## Slide 1
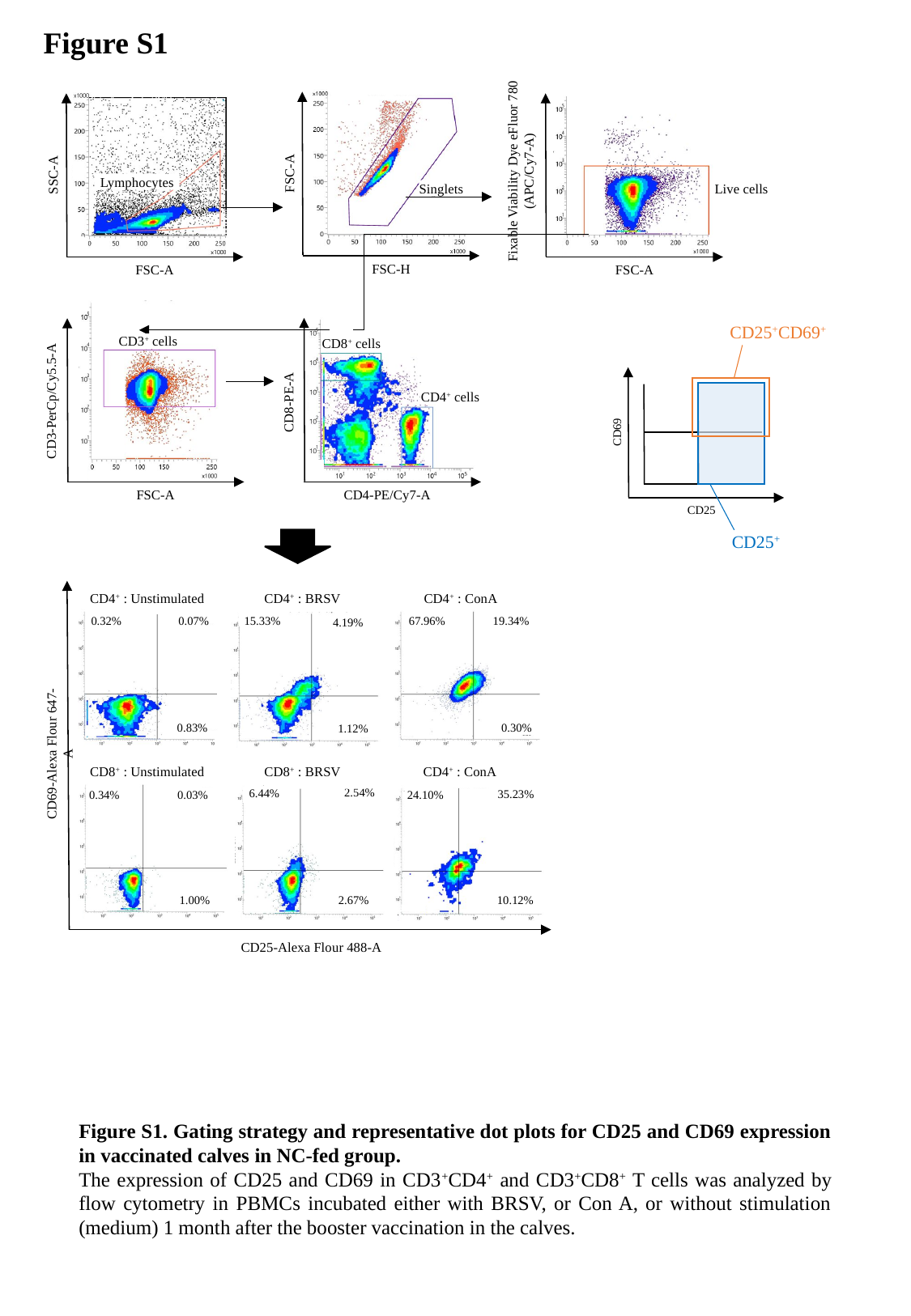

Figure S1
SSC-A
FSC-A
Fixable Viability Dye eFluor 780
(APC/Cy7-A)
FSC-A
Lymphocytes
Singlets
Live cells
FSC-H
FSC-A
CD25+CD69+
CD69
CD25
CD25+
CD8-PE-A
CD4-PE/Cy7-A
CD3-PerCp/Cy5.5-A
FSC-A
CD3+ cells
CD8+ cells
CD4+ cells
CD4+ : Unstimulated
CD4+ : BRSV
CD4+ : ConA
0.32%
0.07%
15.33%
67.96%
19.34%
4.19%
0.83%
0.30%
1.12%
CD69-Alexa Flour 647-A
CD8+ : Unstimulated
CD8+ : BRSV
CD4+ : ConA
2.54%
6.44%
35.23%
0.34%
0.03%
24.10%
1.00%
2.67%
10.12%
CD25-Alexa Flour 488-A
Figure S1. Gating strategy and representative dot plots for CD25 and CD69 expression in vaccinated calves in NC-fed group.
The expression of CD25 and CD69 in CD3+CD4+ and CD3+CD8+ T cells was analyzed by flow cytometry in PBMCs incubated either with BRSV, or Con A, or without stimulation (medium) 1 month after the booster vaccination in the calves.

## Slide 2
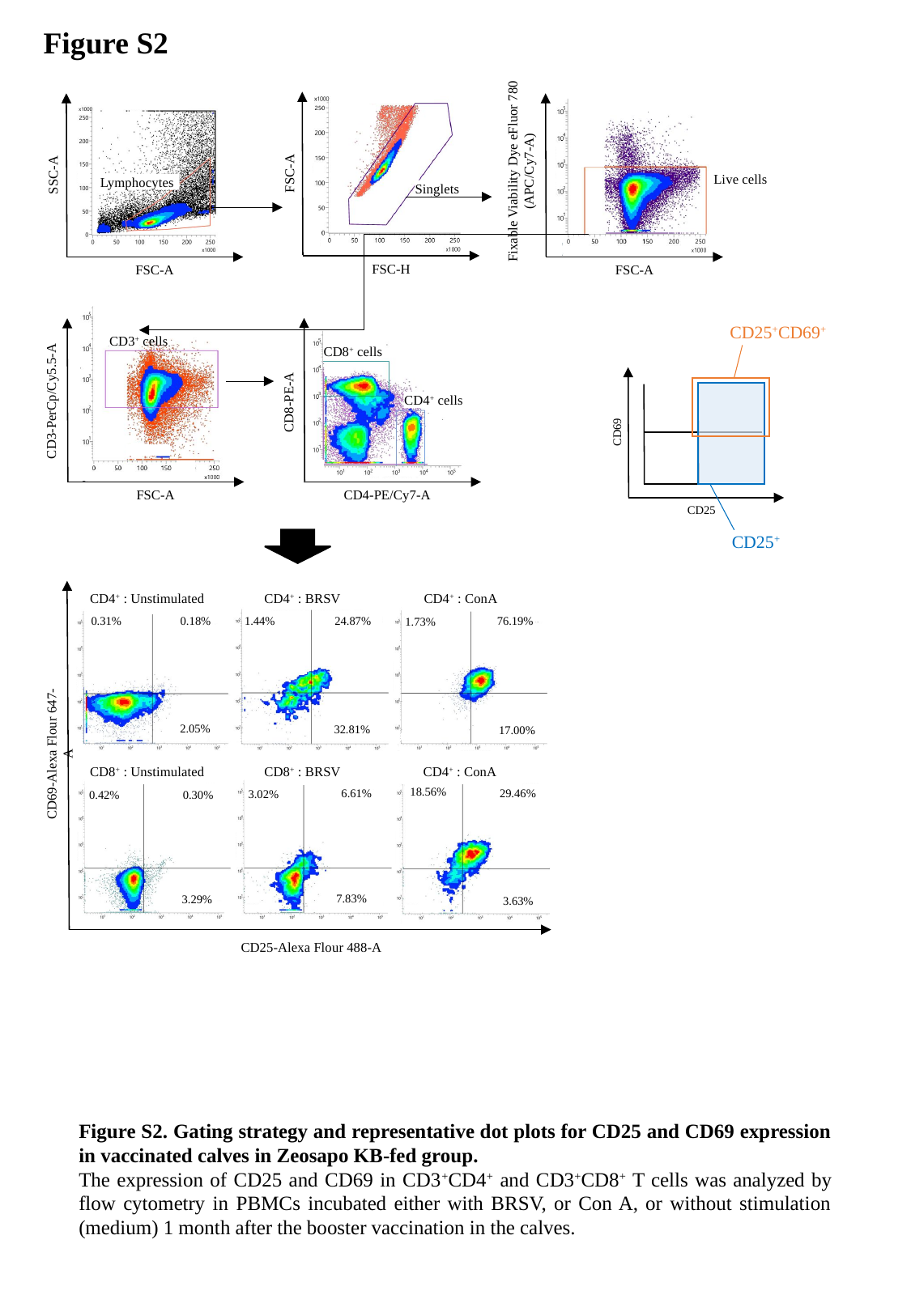

Figure S2
SSC-A
FSC-A
Fixable Viability Dye eFluor 780
(APC/Cy7-A)
FSC-A
Live cells
Lymphocytes
Singlets
FSC-H
FSC-A
CD25+CD69+
CD69
CD25
CD25+
CD8-PE-A
CD4-PE/Cy7-A
CD3-PerCp/Cy5.5-A
FSC-A
CD3+ cells
CD8+ cells
CD4+ cells
CD4+ : Unstimulated
CD4+ : BRSV
CD4+ : ConA
0.31%
0.18%
1.44%
24.87%
76.19%
1.73%
2.05%
32.81%
17.00%
CD69-Alexa Flour 647-A
CD8+ : Unstimulated
CD8+ : BRSV
CD4+ : ConA
18.56%
6.61%
29.46%
3.02%
0.42%
0.30%
7.83%
3.29%
3.63%
CD25-Alexa Flour 488-A
Figure S2. Gating strategy and representative dot plots for CD25 and CD69 expression in vaccinated calves in Zeosapo KB-fed group.
The expression of CD25 and CD69 in CD3+CD4+ and CD3+CD8+ T cells was analyzed by flow cytometry in PBMCs incubated either with BRSV, or Con A, or without stimulation (medium) 1 month after the booster vaccination in the calves.

## Slide 3
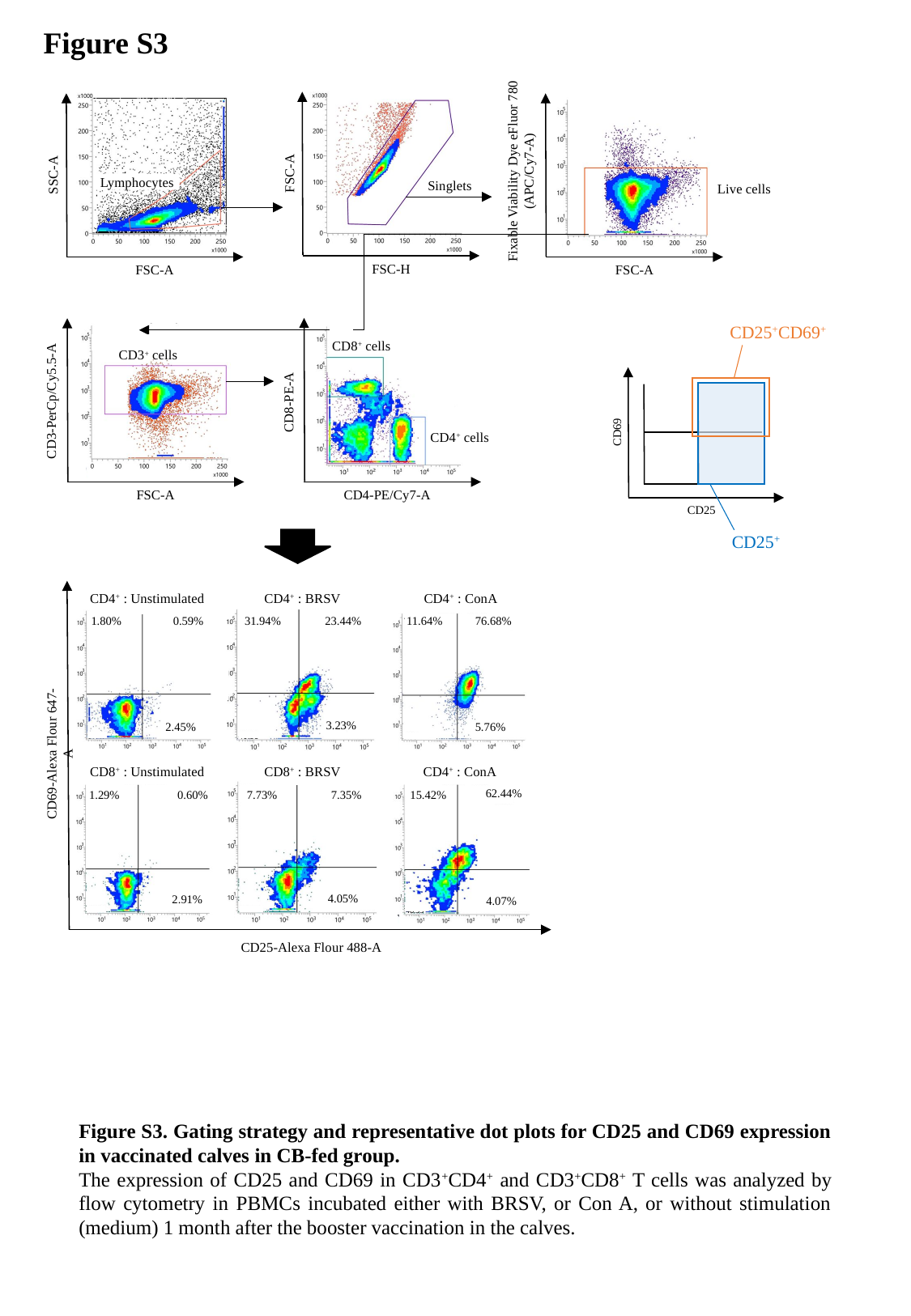

Figure S3
SSC-A
FSC-A
Fixable Viability Dye eFluor 780
(APC/Cy7-A)
FSC-A
Lymphocytes
Singlets
Live cells
FSC-H
FSC-A
CD25+CD69+
CD69
CD25
CD25+
CD8-PE-A
CD4-PE/Cy7-A
CD3-PerCp/Cy5.5-A
FSC-A
CD8+ cells
CD3+ cells
CD4+ cells
CD4+ : Unstimulated
CD4+ : BRSV
CD4+ : ConA
1.80%
0.59%
31.94%
23.44%
11.64%
76.68%
3.23%
2.45%
5.76%
CD69-Alexa Flour 647-A
CD8+ : Unstimulated
CD8+ : BRSV
CD4+ : ConA
62.44%
1.29%
0.60%
7.73%
7.35%
15.42%
4.05%
2.91%
4.07%
CD25-Alexa Flour 488-A
Figure S3. Gating strategy and representative dot plots for CD25 and CD69 expression in vaccinated calves in CB-fed group.
The expression of CD25 and CD69 in CD3+CD4+ and CD3+CD8+ T cells was analyzed by flow cytometry in PBMCs incubated either with BRSV, or Con A, or without stimulation (medium) 1 month after the booster vaccination in the calves.
